# Opportunistic Large Array Propagation Models: A Comprehensive Survey

**DOI:** 10.3390/s21124206

**Published:** 2021-06-19

**Authors:** Farhan Nawaz, Hemant Kumar, Syed Ali Hassan, Haejoon Jung

**Affiliations:** 1 School of Electrical Engineering & Computer Science (SEECS), National University of Sciences & Technology (NUST), Islamabad 44000, Pakistan; fnawaz.msee16seecs@seecs.edu.pk (F.N.); hkumar.msee16seecs@seecs.edu.pk (H.K.); ali.hassan@seecs.edu.pk (S.A.H.); 2 Department of Information and Telecommunication Engineering, Incheon National University, Incheon 22012, Korea

**Keywords:** 5G, B5G, massive machine-type communications (mMTC), massive Internet-of-Things (IoT), Opportunistic Large Array (OLA), cooperative transmission (CT), propagation modeling, node density

## Abstract

Enabled by the fifth-generation (5G) and beyond 5G communications, large-scale deployments of Internet-of-Things (IoT) networks are expected in various application fields to handle massive machine-type communication (mMTC) services. Device-to-device (D2D) communications can be an effective solution in massive IoT networks to overcome the inherent hardware limitations of small devices. In such D2D scenarios, given that a receiver can benefit from the signal-to-noise-ratio (SNR) advantage through diversity and array gains, cooperative transmission (CT) can be employed, so that multiple IoT nodes can create a virtual antenna array. In particular, Opportunistic Large Array (OLA), which is one type of CT technique, is known to provide fast, energy-efficient, and reliable broadcasting and unicasting without prior coordination, which can be exploited in future mMTC applications. However, OLA-based protocol design and operation are subject to network models to characterize the propagation behavior and evaluate the performance. Further, it has been shown through some experimental studies that the most widely-used model in prior studies on OLA is not accurate for networks with networks with low node density. Therefore, stochastic models using quasi-stationary Markov chain are introduced, which are more complex but more exact to estimate the key performance metrics of the OLA transmissions in practice. Considering the fact that such propagation models should be selected carefully depending on system parameters such as network topology and channel environments, we provide a comprehensive survey on the analytical models and framework of the OLA propagation in the literature, which is not available in the existing survey papers on OLA protocols. In addition, we introduce energy-efficient OLA techniques, which are of paramount importance in energy-limited IoT networks. Furthermore, we discuss future research directions to combine OLA with emerging technologies.

## 1. Introduction

Massive machine-type communications (mMTC) is one of the major service scenarios in fifth-generation (5G) mobile communication systems, where a large number of Internet-of-Things (IoT) nodes are expected to deployed in various fields of application such as environment monitoring, building management, and industrial networks [1,2,3]. In massive IoT networks, the devices generate a significant number of data, which can be exploited to streamline operational processes and develop more innovative product and services across various industries [4]. In this context, for real-time collection, processing, and analysis of the massive IoT data, it is essential to better design and operate communication protocols, since the communication overhead is considerable [5]. Furthermore, mMTC for IoT data collection should be handled in the presence of the inherent limitations in IoT devices such as hardware and power limitations.

Device-to-device (D2D) communication, which allows direct local communication between devices, can be a powerful means to meet the requirements of mMTC services without a heavy signaling overhead, as noted in [6]. Furthermore, combined with node cooperation, D2D can achieve better reliability and spectral efficiency [7,8]. Especially, with a large-scale deployment of IoT nodes, the node cooperation becomes more beneficial to improve data rate [9] and information security [10].

For the aforementioned reasons, in this paper, we consider cooperative transmission (CT) techniques as a solution to overcome the hardware limitations of IoT nodes, where one or more devices transmit the same message using a diversity of channels. In the CT networks, a receiver can combine multiple copies of the same message signals, which gives a signal-to-noise-ratio (SNR) advantage compared to conventional non-CT techniques. Such a SNR advantage can be used for range extension and improvements in reliability, energy efficiency, and latency [11]. In a experimental study of CT in [12], it was shown that the transmission range can be significantly extended, while non-CT schemes suffer from the highly limited coverage of the IoT nodes. Furthermore, CT can provide more reliable communication in the presence of multi-path fading and shadowing by constructing virtual multiple-input-single-output (VMISO) links [13]. This improvement in reliability through diversity and array gains can be realized using a collocated (or real) antenna array, but CT is more desirable in the IoT networks, due to the space and power limitations of small devices to accommodate the multiple antennas.

Opportunistic Large Array (OLA) is a fast and promising CT technique, where a message signal hops from one layer of nodes to another [14]. At first, a source node broadcasts a message signal, which is received by the nodes near the source. The nodes that receive the source message can participate in forwarding the message to the next hop in CT, which can be determined based on the protocol design. This adaptive relaying can be realized in decode-and-forward (DF) or amplify-and-forward (AF) methods [15], but the DF protocol is more widely used in practice. In the case of the DF relaying, all the nodes that receive the message signal from the source try to decode the message. The nodes that successfully decode the message are called DF nodes. In OLA, the DF nodes relay the same message signal from the source together, shortly after reception, without coordination with each other. In the following hops, this process continues until the message signal reaches the destination node or broadcasted over the entire network. In other words, each node that receives the message directly from the source becomes a member (or part) of OLA 1 (or level 1). Some of the OLA 1 nodes that decode the message signal without any error will further relay it to the next hop. Then, the nodes that receive the message signal from OLA 1 will become the part of OLA 2 (or level 2), which again forward the message to the next hop in CT.

The group transmissions of the multiple relays in OLA can be synchronized based on the packet reception [14], while conventional networks may suffer from frequent collisions without precise network synchronization due to random channel access of multiple nodes (e.g., carrier-sense multiple access or CSMA). The experimental results using software-defined radio (SDR) test-bed in [16] show that 90% of the transmit time spread is less than 300 ns for OLA networks with 10 hops. Therefore, OLA multi-hop transmissions can be collision-free without coordination.

In OLA transmissions, spatial diversity can be achieved at a receiver by applying a diversity of combining techniques such as maximum ratio combining (MRC), equal gain combining (EGC), or selective combining (SC) on the multiple copies of the same message signal. On the top of that, the avalanche of the incoming signals produces a stronger signal at the receiver, which corresponds to the array gain. The SNR advantage through both diversity and array gains can help reach a distant destination with a lower number of hops in an energy-efficient manner [17]. It is noted that OLA can be easily implemented on any network or system, because it is compatible with any modulation techniques at the transmitter and a receiver that uses a variety of detection techniques for successful detection of the message.

**Figure 1:**
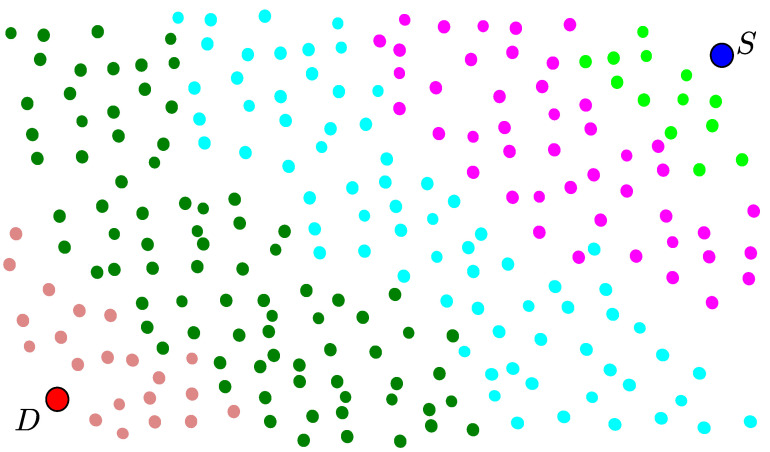
An example illustration of multi-hop OLA transmissions.

Figure 1 shows an example illustration of multi-hop OLA transmissions from the source, which is indicated by the blue circle, to the destination, which is represented by the red circle. Furthermore, the smaller circles represent IoT nodes, which participate in the OLA propagation as relays. The different colors in the figure correspond to different OLA memberships (i.e., levels). The source cannot directly communicate or send message information to the destination node because of high path loss and limited energy as an IoT node. Thus, the other nodes, which are randomly placed in between the source and the destination, forward the source message in multi-hop OLA transmissions. The source broadcasts the message signal, which is received by the neighboring relay nodes, indicated by the light green circles. These nodes will try to decode the message and forward it to the next adjacent nodes in the next hop in the next time slot. This process continues until the message reaches the destination node in the next few hops. During such consecutive OLA transmissions, the participating nodes become members of OLA *n* (or level *n*) without requiring prior coordination among relay nodes, as shown in the figure.

The well-known advantages of the OLA networks are range extension [12], simple and inexpensive routing [18], medium access control (MAC)-free broadcasting [19], and energy efficiency [20,21]. OLA and its variants are extensively used and studied in cognitive networks [22], MIMO mobile ad hoc networks [23], security for IoT networks [24], healthcare monitoring [25,26], and routing strategies [27]. Owing to its effectiveness, a significant amount of work has been made on the OLA protocols to better exploit such advantages. However, for analytical simplicity, most of the existing studies, including [14,17,18,19,20,21,28,29,30,31,32,33,34], perform analysis using an approximated network model, which is referred to as *continuum assumption*, without considering random channel effects, which can be accurate enough for high node density networks. However, in practice, the authors of [12,35,36] observe considerable performance gaps between their measurement and the theoretical analysis, assuming the simplified models, when the node density is not high enough. To overcome such restriction in the prior model, the authors of [37] first propose a more realistic linear network model that can work well even with the low node density using quasi-stationary Markov chains. At the same time, they consider multi-path fading and quantify the diversity gain in various channel environments. In the following studies, OLA network models with different topologies are proposed for various applications.

In this context, for more precise analysis and performance evaluation of OLA-based protocols, it is critical to establish a proper OLA network model depending on system parameters and channel environments. Further, as a fundamental factor to select and operate OLA schemes, reviewing existing network models is of radical importance, which can also affect the future of massive IoT technology. To this end, the main purpose of this review paper is to provide a reference to better choose the most suitable theoretical framework for OLA networks, which will allow a better strategy to realize 5G innovations in the mMTC scenario. We note that this paper is distinct from a prior survey of OLA techniques in [18,38], because this paper is the first comprehensive survey of OLA network models. Specifically, the authors of [18] present the OLA broadcasting and unicasting methods assuming high node density. This survey is outdated, since it was published in 2008. For this reason, they do not include finite-density OLA network models. The second survey in [38] includes energy balancing and MAC protocols for OLA networks. However, most of the studies discussed in [38], which was published in 2015, assume deterministic channel assumptions, which highly limits the applicability in realistic channel environments with multi-path fading. In addition, both [18,38] do not cover stochastic analysis of OLA propagation based on finite-density models proposed in [37,39,40,41,42,43,44,45,46,47,48]. We note that the finite-density models, which are extensively discussed in this paper, can provide better guidelines on the OLA protocol design and operation in practice. The OLA techniques presented in this paper can be exploited in wireless sensor networks, IoT, communications and signal processing [49,50,51,52,53,54,55], which are representative topics covered in Sensors. Furthermore, the OLA protocols can be employed in small sensors to improve their energy efficiency.

The rest of the paper is organized as follows. In Section 2, we consider the continuum model, in which the node density is assumed to be infinite. In Section 3, Section 4 and Section 5, we present OLA network models with finite node density under random fading channels. Specifically, one-dimensional (1D) linear models are introduced in Section 3, while two-dimensional (2D) strip network models are discussed in Section 4. In addition, the theoretical framework to analyze intra-flow interference caused by co-channel multi-packet transmissions is considered in Section 5, which is important in throughput evaluation. In Section 6, we present energy-efficient OLA protocols, which are suitable for IoT networks. Furthermore, future research directions of OLA are provided in Section 7. Finally, the paper is concluded in Section 8.

## 2. Continuum Model with Deterministic Channel Assumption

Table 1 shows the summary of existing OLA propagation models for single-packet transmission, which are distinguished by shape, density, node locations, set boundary, and the number of sources. In this section, we first consider models with the infinite node density assumption. Then, in Section 3 and Section 4, we introduce the one-dimensional and two-dimensional models with finite density, respectively.

For multi-hop networks with high node density, continuum assumption is widely employed, as in [14,17,28,29]. In this model, the node density ρ is assumed to be very high (i.e., ρ→∞), while the transmit power of relays per unit area, which is denoted by ${\bar{P}}_{r}=\rho P_{r}$, is treated to be constant. Therefore, this approximation becomes more exact, as the node density or degree increases.

In addition to the continuum model, which simplifies the random locations of nodes, a deterministic channel assumption is also assumed in various studies on OLA-based protocols such as OLA broadcasting [21,30], OLA unicasting [18,31,32], energy-efficient schemes [20,21,33], and multi-packet OLA transmissions [19,34]. Based on this deterministic channel assumption, random channel effects such as multi-path fading and shadowing are not considered, which provides analytical simplicity. However, from realistically, the continuum model, which assumes the infinite node density, has limited applicability with the deterministic channel assumption. For example, the OLA propagation characteristics in low-density networks do not follow the continuum model. Furthermore, the diversity gain in fading channels cannot be reflected appropriately using the deterministic channel assumption. More realistic models that provide the solid theoretical framework in such cases will be discussed in Section 3 and Section 4.

**Figure 2:**
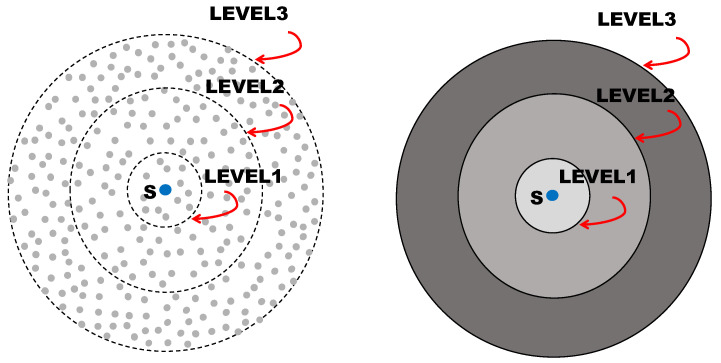
Continuum approximation of a dense *disk* network.

Figure 2 shows a disk-shaped dense network (the left figure) and its continuum model (the right figure). In this model, all the nodes are uniformly and randomly distributed over a circular disk-shaped network. The source is located at the center of the disk as indicated by the blue circle labeled with “S” in the figure. The gray-colored concentric rings represent the OLAs for each level of decoding, which are created by consecutive relaying of the decoded message. In this manner, the message signal can be broadcast to the entire network with significantly lower latency compared to non-CT protocols such as flooding. In [21], the energy efficiency of the OLA broadcasting is improved up to 32% by letting only the nodes in an outer circular area to relay, excluding the nodes in the inner circular area at the OLA level.

**Figure 3:**
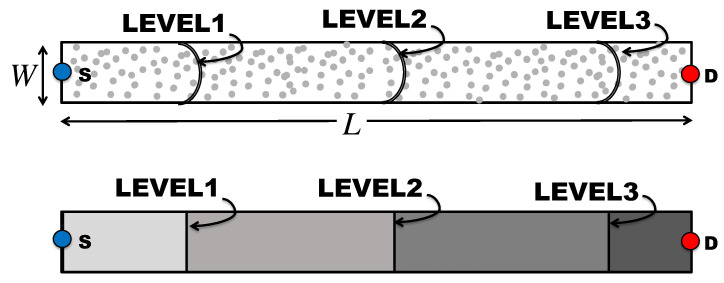
Continuum approximation of a dense *strip* network.

Figure 3 depicts a dense strip network for OLA-based unicasting and its continuum model. This strip continuum network is an idealized model of a cooperative route constructed by OLA-based routing protocols proposed in [18,31,32]. In this model, all the nodes are distributed uniformly over rectangular areas which correspond to different OLA levels. As in the disk network, an energy-efficient protocol is proposed in [20] by exploiting disparate path losses to the nodes in the next level.

In both models with the continuum and deterministic channel assumptions, there is no uncertainty in network topology as well as OLA boundaries. As a result, the propagation characteristics such as hop distance, latency, and energy consumption are always deterministic. Thus, it is straight-forward to analyze impacts of various system parameters such as node density, transmit power, and path-loss exponent. However, as expected, this model is accurate only when the node density of the network is considerably high, which cannot be satisfied in practical scenarios. For example, experimental results with software-defined radio (SDR) test-bed in [12,35,36] show stochastic propagation behaviors and significant discrepancy from the theoretical analysis based on the continuum network model. In addition, the authors of [56,57] show that the impact of path-loss disparity subject to the random network topology on outage probability can be approximated by log-normal distribution with a large standard deviation. Barrage relay network (BRN), another branch of cooperative flooding in a controlled fashion, has also been studied to a large extent as a type of ad hoc network that uses cooperation at each hop. Many variants and applications of BRNs have been studied such as unicasting [58], simulation analysis [59], capacity analysis [60], multi-source broadcast [61], cooperative transport [62], and protocol design [63].

## 3. One-Dimensional Propagation Models of OLA-Based Networks

In this section, we first consider one-dimensional (1D) OLA network models with finite node density. Unlike the continuum model, which has uniform power density over the entire network, the transmit power is clustered at a finite number of nodes in these models. Therefore, these finite-density models can provide more realistic and accurate performance evaluation of OLA protocols especially with low node density.

### 3.1. Non-Overlapping 1D OLA Network

**Figure 4:**
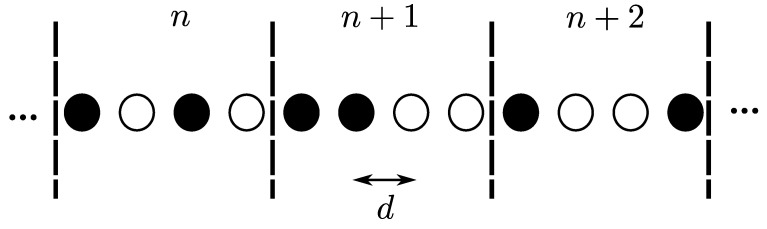
Non-overlapping 1D OLA network.

In this network model, which is proposed in [37], a finite number of nodes are arranged along a straight line with uniform inter-node separations, as shown in Figure 4. This uniform 1D network can be considered as a multi-hop route along a linear structure such as hallways and bridges. In the figure, nodes are divided into sets, the boundaries of which are indicated by the dotted lines. Each set consists of a fixed number of nodes, which is four in the example in Figure 4, which are entitled to be the same OLA level (membership) or hop. In this model, different sets (groups) are deployed in a non-overlapping manner. In other words, a node cannot be a member of multiple sets. However, even with such deterministic groups, actual OLA membership of each node is opportunistic in a binary manner, because only the nodes in the cluster that can decode the message will be part of it, while the nodes that fail to decode cannot participate in the group transmission.

The first key parameter of this model is the distance between the adjacent nodes, which is denoted by *d* in the figure. Furthermore, the second key parameter of the multi-hop OLA transmissions along the 1D linear network is the number of nodes in each group that determines which nodes are qualified to relay the message signal together to the next hop. As in [37], at any OLA level (or hop), a node is considered to decode successfully, when its received SNR from the prior level is greater than or equal to the decoding threshold. In the example illustrated in Figure 4, the black circles are the nodes that successfully decoded the signal, while the white circles represent the nodes that fail to decode. Thus, only the nodes indicated by the black circles can participate in OLA transmissions to the next hop.

In a multi-hop OLA network, because each node receives multiple copies of the same message signal from multiple nodes in the previous level, it can achieve both array and diversity gains. As a result, the message can be transmitted more reliably to the destination, which is unreachable directly from the source, compared to conventional SISO multi-hop transmissions. Furthermore, by the range-extension property of CT, OLA takes less hops, as compared to non-CT protocols. Further, even compared to other CT schemes, OLA provides lower end-to-end latency, because OLA nodes immediately broadcast (or forward) the data to adjacent nodes when they successfully decode them without any coordination with other nodes. In this way, OLA is also energy-efficient, because only nodes that have decoded the message signal correctly will be part of the level, while the other nodes will save their energy.

### 3.2. Overlapping 1D OLA Network

**Figure 5:**
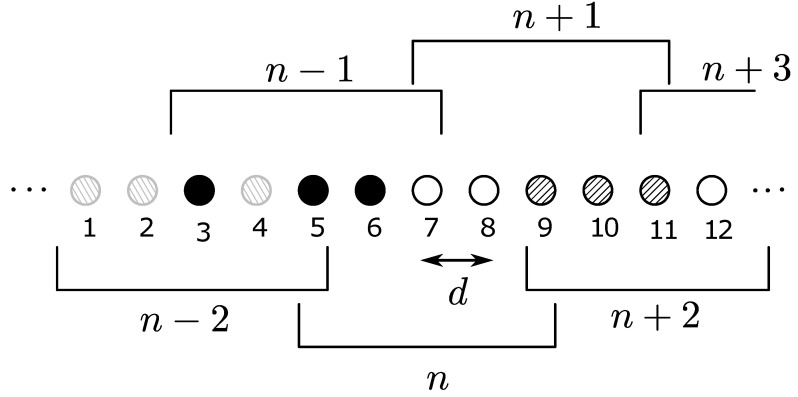
Overlapping 1D OLA network.

In this network model, nodes are placed in the same way as in the non-overlapping case. However, in this model, the *m*th node can be a member of different OLAs in a region of support based on the hop count that it can decode the message for the first time. In other words, as shown in Figure 5, the *n*th set of nodes have overlapping windows with the (n−2)th, (n−1)th, (n+1)th, and (n+2)th sets. For example, the node $m_{7}$ can have an OLA membership level from (n−2) to (n+2), depending on the hop count when it first decodes.

In the figure, window size, which is the number of nodes in each set, is set to be five, while a shift in window, which corresponds to the number of nodes that are not part of the previous adjacent set, is assumed to be 2. In this example, nodes $m_{1}$, $m_{2}$, and $m_{4}$ in the (n−2)th set become the part of OLA, because they decode the message successfully. Now, these nodes will forward the message to the next adjacent set in next time slot. Only the nodes in the (n−1)th OLA may decode that message, but since $m_{4}$ has already taken part in OLA (n−2), it will not participate in any other OLA, including (n−1). Only $m_{3}$, $m_{5}$, and $m_{6}$ successfully decode the message in OLA (n−1) and this process continues. As presented in [39], this network gives higher energy efficiency compared to the non-overlapping case, because some nodes do not participate in the next OLA if they already became the part of the previous OLA. However, the overall performance will degrade if all the last three nodes in OLA (n−2) take part in it and do become the part of OLA (n−1). It is also possible that the last two nodes of OLA (n−1) do not decode the message; hence, no node will relay the message in OLA (n−1).

The received power at the *j*th node at time instant n+1 is given by

(1) $$P_{r_{j}}\left(n+1\right)=\frac{P_{t}}{d^{\beta }}\sum_{m\in N_{n}}\frac{\mu_{mj}}{|h_{d}{-m+j|}^{\beta }},$$

where the summation is over the nodes that were decoded correctly in the previous level. The flat fading Rayleigh channel gain from node *m* in the previous level to node *j* in the current level is denoted by $\mu_{mj}\in \mu$; the elements of μ are independently and identically distributed (i.i.d.) and are drawn from an exponential distribution with the parameter $\sigma_{\mu }^{2}$ = 1; β is the path loss exponent with a usual range of 2–4. Consequently, the received SNR at the *j*th node is given as $\gamma_{j}=P_{r_{j}}/\sigma_{j}^{2}$, where $\sigma^{2}$ is the variance of the noise in the receiver. The probability of outage for a node *j* is given as $1-P\left\{\gamma_{j}>\tau \right\}$, where

(2) $$P\left\{\gamma_{j}>\tau \right\}=\int_{\tau }^{\infty }p_{\gamma_{j}}\left(y\right)dy.$$

$p_{\gamma_{j}}\left(y\right)$ is the probability density function (PDF) of the received SNR at the *j*th node, and τ is the modulation dependent threshold. A detailed derivation of the PDF is given in [39], and the results show that an increase in transmit power increases the success probability at a node and hence the probability of message traversal to longer distances increases.

### 3.3. 1D OLA Network with Co-Locating Groups of Nodes

**Figure 6:**
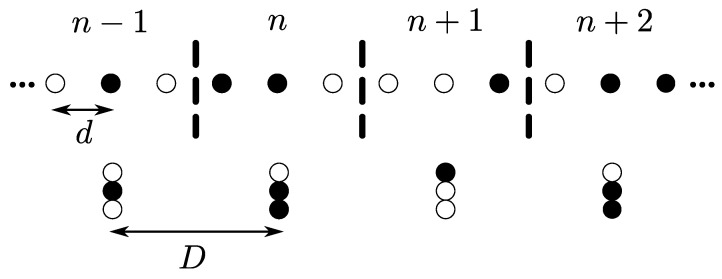
1D OLA network with co-locating groups of nodes.

In this network topology, groups of collocated nodes are equally spaced on a horizontal line and partitioned by the vertical dashed lines as shown in Figure 6. The groups (or sets) are labeled as n−1, *n*, n+1, n+2, and so on. While all of the nodes in the same group are collocated, the inter-group distance is *D*. Each of multi-hop transmissions occurs if there exists at least one node in this OLA level that decodes the received message from the previous level and then relays the same message to its next level. This process is repeated in a sequential manner in the following hops until the message reaches the destination. The difference between the non-overlapping network topology and this topology is that, in the non-overlapping scenario, cooperators have different path losses, while they have the same path loss in the collocated scenario. Furthermore, in the non-overlapping nodes topology, as shown in Figure 6, the average power gathered at a single point from dispersed nodes is much higher than from a group of collocated nodes topology, which is placed at the centroid position.

Since all the nodes are collocated, and there are no disparate path losses that affect the parameter of the exponential distribution, the PDF of the received power at the *k*th node in a cluster is Gamma distribution given as [40]

(3) $$p_{\gamma_{k}}\left(y\right)=\frac{1}{(K_{n}-1)!}{\tilde{\lambda }}^{K_{n}}y^{\left(K_{n}-1\right)}\exp(-\tilde{\lambda }y).$$

Evaluating (2) to get the conditional success of the *k*th node, we have

(4) $$P\left\{\gamma_{k}\left(n\right)>\tau \right\}=\frac{1}{(K_{n}-1)!}\Gamma \left(K_{n},\tilde{\lambda }\tau \right),$$

where $\Gamma (K_{n},\tilde{\lambda }\tau )$ is the upper incomplete Gamma function. In [40], it was shown that the outage probability in the collocated scenario is better compared to that of the non-overlapping scenario with independent Rayleigh fading for the members in the same sets. Further, it has been mathematically proven in [40] that the collocated 1D network topology provides better coverage of the network compared to the one dimensional non-overlapping network topology, but the degree of improvement depends on the path-loss exponent and other channel parameters.

### 3.4. Randomly Deployed 1D OLA Network

**Figure 7:**
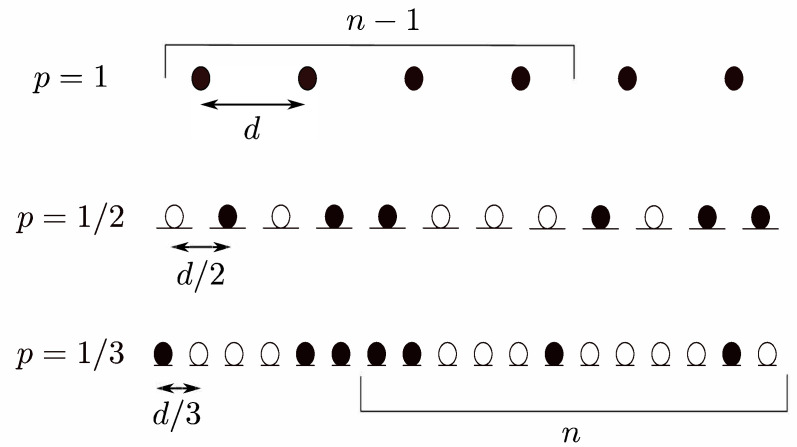
1D OLA network with random node deployment.

The network topology is considered 1D with fixed density, in which the nodes are randomly located as shown in Figure 7, using a Bernoulli random process. In particular, this deployment is considered as a case study where locations of potential node are equally distant from their adjacent nodes but the absence or presence of any node at any location is according to the Bernoulli random process. That is, for every node location, a random variable *B*, which follows the Bernoulli distribution, has an outcome B=0 with probability 1−p, if node is absent, and B=1 with probability *p*, if the node is present. In Figure 7, different linear networks with different extent of randomness but same average density of candidates is shown. The three stacked linear networks in Figure 7 show examples of possible Bernoulli deployments with p=1, 1/2, and 1/3, respectively. The node placements are integer multiples of *d*, where *d* is the distance between adjacent nodes in the 1D networks. The black circles denote the presence of the node, while the white circles indicate the absence of nodes. The levels are labeled as n−1, *n* …, etc., and a hop occurs when nodes in any level transmit the message in forward direction and at least one node in the next level can decode the message. The success probability in the end-to-end packet delivery from the source to the destination is simply the product of all links’ success probabilities, which means that end-to-end success probability is significantly lower than that of individual link success probability, when there are a large number of hops. As investigated in [41], random deployments of nodes cause an SNR disadvantage compared to deterministic deployments for the same probability of success.

## 4. Two-Dimensional Propagation Models of OLA-Based Networks

In this section, we consider two-dimensional (2D) propagation models, where nodes are placed along both vertical and horizontal dimensions, which can be an idealized version of OLA-based routes with more node participation compared to the 1D linear networks [38].

### 4.1. Overlapping 2D OLA Network

**Figure 8:**
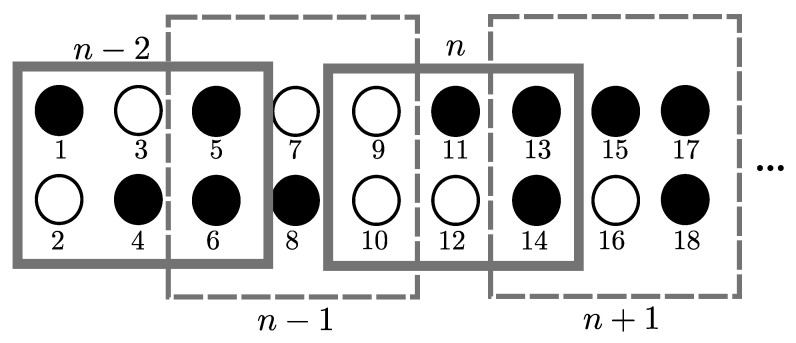
Overlapping 2D OLA network.

In [42], the authors assume a 2D grid network with an infinite length and a fixed width in the horizontal and vertical dimensions, respectively. Figure 8 shows an example topology where the separation between any two adjacent nodes is *d* in both dimensions. The levels or OLAs are labeled as n−2, n−1, *n*, n+1, etc. Each set has a rectangular shape of the area with a window size *M*, which corresponds to the number of nodes in each set. In Figure 8, M=6, where L=3 and W=2, which correspond to the numbers of nodes in the horizontal and vertical axis in each set. The shift in window (or hop-distance) is 2 in the horizontal dimension. The nodes in a level are indexed from top to bottom and then left to right. In level n−2, nodes 1, 4, 5, and 6 can decode the message. In the next time slot, these nodes relay the message only to the nodes in the n−1th level, and only these nodes may decode the message. In level n−2, two nodes (5 and 6) have already taken part; therefore, they cannot participate in level n−1 or any other level. Thus, nodes 7, 8, 9, and 10 become the candidate nodes of level n−1 and only node 8 becomes a DF node, and this process continues. The smaller the number of nodes in a level, the larger the SNR margin required to reach the far distances for the same hop distance. For this 2D network topology, applying finite node density is straightforward, because the locations of candidate nodes are known, and there is no uncertainty in the presence or absence of nodes in a specific area [42].

### 4.2. Non-Overlapping Fixed Boundary 2D OLA Network with a Fixed Number of Nodes at Arbitrary Locations

**Figure 9:**
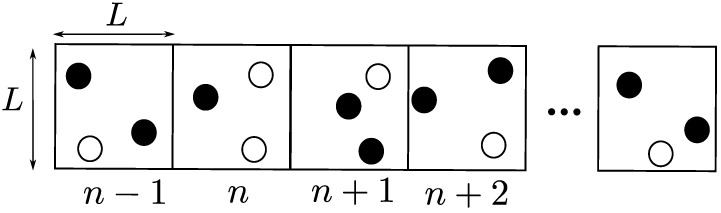
Non-overlapping fixed boundary 2D OLA network with a fixed number of nodes at arbitrary locations.

In Figure 8, the network topology is two dimensional, but the locations of nodes are fixed in any OLA, which limits the practical value of the model. In other words, in reality, nodes can be located randomly, which are also not always known to other nodes in the network. Therefore, in this model, illustrated in Figure 9, the strip-shaped network is divided into contiguous square regions, in which nodes are randomly placed. Each square is labeled with its level or OLA membership (i.e., n−1, *n*, and so on). It is assumed that the node density is the same for each OLA. The boundaries are confined, and transmission from the nodes of one OLA is for the nodes of the adjacent OLA only. The notion of fixed boundary is to reduce the complexity of geometry of usual OLA network topologies, which do not have symmetrical levels in every realization. This constraint is considered because, for many strip-shaped networks, cooperating nodes of two adjacent levels form disjoint sets, as in [43]. This model introduces random nodes placed in fixed square regions in a horizontal contiguous strip-shaped network to find the coverage. The nodes at OLA n−1 transmit the message signal to OLA *n*, and the nodes at OLA *n* make use of a diversity-combining technique and try to decode the message coming from OLA n−1. In Figure 9, only one node in OLA *n*, which is indicated by the black-filled circle, could decode the message signal. This process continues until the message signal reaches its destination node at a certain OLA. A hop occurs if at least one of any nodes of OLA can decode the message. This network topology, which has fixed boundary, is opportunistic with the meaning of that any node at a certain level. For example, at OLA *n*, node(s) can or cannot decode the message coming from OLA n−1, because both channel conditions and node positions are random.

### 4.3. Non-Overlapping Fixed Boundary 2D OLA Network with a Random Number of Nodes with Arbitrary Locations

**Figure 10:**
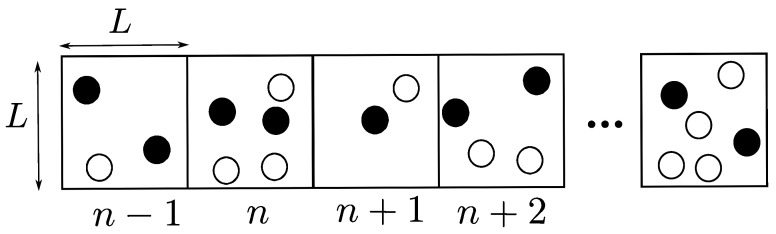
Non-overlapping fixed boundary 2D OLA network with a random number of nodes at arbitrary locations.

This network topology, which is proposed in [44], is almost the same as the previous model shown in Figure 9, but the difference is that there are an arbitrary random number of nodes at each level, i.e., different numbers of nodes for different levels. Each square shown in Figure 10 corresponds to a level, and each level contains different numbers of nodes having random locations in a 2D strip-shaped network. For illustration, the hop occurs when the nodes at the leftmost square, level n−1, receive the message signal from any source node or previous OLA, and then relay that signal to the level *n*, in the next time slot, which is the same as the OLA transmissions in the previous models. Each node applies a diversity-combining technique such as MRC, EGC, and SC on multiple received copies of the same signal. The black-filled circles are called DF nodes that successfully decode the message received from the previous OLAs. This network topology is more practical compared to the previous model with a fixed number of nodes in each OLA, because it allows a different number of nodes in each hop to decode the message signal received. In both models, the probability of successful hop is subject to channel condition and locations of nodes, which are random. However, by allowing different numbers of nodes in each OLA, this model can be also used to consider networks with network partitions in intermediate hops.

### 4.4. Large-Scale 2D OLA Network with Random Boundaries

As hop boundaries are also not deterministic in practice, the authors in [45] propose a model, where both number of nodes in any hop and OLA boundaries are random. Thus, this model can be used to characterize OLA transmissions with inherently irregular hops or levels with random nodes in each level, as the source transmits a signal to a distant destination, which, with the help of typical OLA protocol. Since the nodes are randomly located and hop boundaries are also randomly determined, path loss also becomes random. For CT, multiple SISO links from corresponding nodes of any level become the VMISO links for the node of next level. The modeling of node locations is done as follows. We let the total length of the network be divided into multiple hops, such that the average hop distance is denoted by μ. Let ϕ denote a homogeneous Poisson point process (PPP) on a hop with intensity $\tilde{\lambda }$ such that the average number of nodes in a hop is $\tilde{\gamma }=\tilde{\lambda }\left|S\right|$, where |S| denotes the area of a single hop. The probability of having $k_{n}$ nodes in one hop is thus given by [45]

(5) $$P\left(\phi \left(S\right)=k_{n}\right)=\exp\left(-\tilde{\lambda }|S|\right)\frac{(\tilde{\lambda }{\left|S|\right)}^{k_{n}}}{k_{n}!}.$$

Thus, the nodes are located as PPP and average power calculations on each of the nodes is performed to find the outage probability.

**Figure 11:**
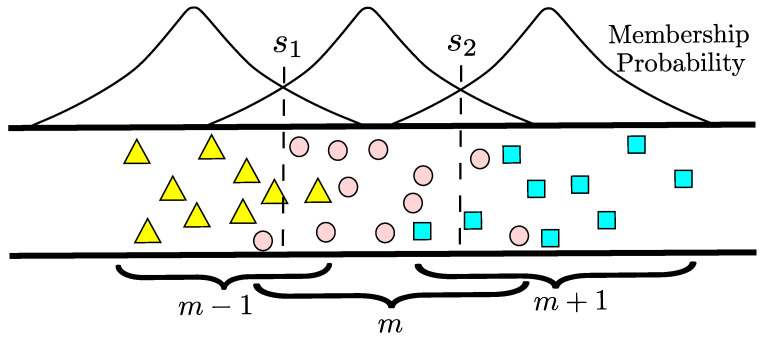
2D strip network with random node locations and membership probability.

The random node locations with finite node density in a strip-shaped 2D are shown in Figure 11, where nodes are uniformly distributed, as indicated by the triangles, circles, and squares, which corresponds to levels m−1, *m*, m+1, respectively. An opportunistic hop or level is made when a set of DF nodes that decode the message signal successfully, which was transmitted by a source node or a set of nodes in the previous level. In the next time slot, the same DF nodes in a level relay the same message signal to the nodes ahead and the next level is formed. Specifically, in the beginning, the source node transmits the message signal that is received by the set of nodes in the vicinity, and out of those, few nodes decode that message signal successfully, depending upon nodes density, transmit power, and decoding threshold. This subset of nodes that decode the message signal successfully become members of the first hop or level. The nodes of level 1 relay the same message signal cooperatively in the next time slot, which forms level 2. This process continues and other subsequent levels are made until the message is broadcast to the entire network or reaches its destination. A hop is formed opportunistically during the entire transmission process, and there are no fixed boundaries between the nodes of two levels, as shown in Figure 11.

**Figure 12:**
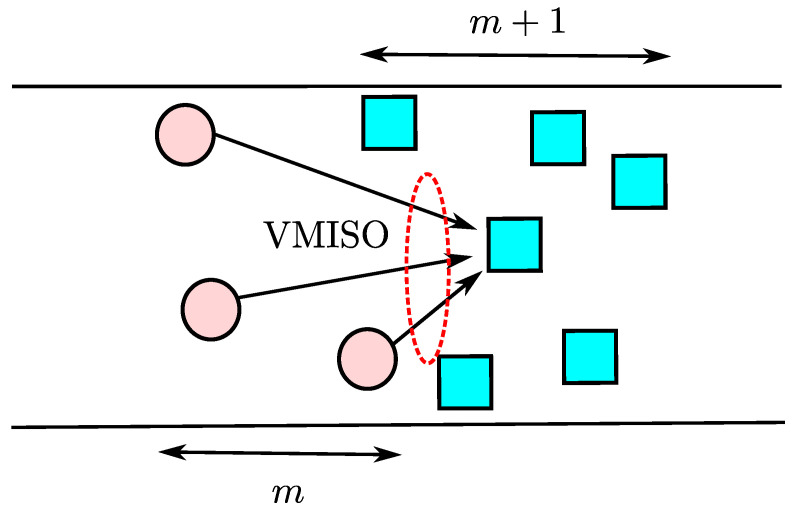
An example illustration of a virtual multiple-input–single-output (VMISO) link.

In such OLA transmissions, VMISO links can be created in each hop, as shown in Figure 12, which provides higher reliability compared to SISO links through spatial diversity. A hop is called successful when at least one DF node exists in the next hop to relay the message signal ahead. A node can be a member of different levels or many levels in different trials, because of randomness in hop boundaries, node locations, and channel characteristics. The tendency of the nodes to be in the same level in multiple iterations of cooperative transmission is lower if the nodes are located near the imaginary boundary of two levels, and tendency increases when nodes are present around the center of a level because of path loss. The nodes located away from the imaginary boundary of levels or around the center of level cannot become the part of adjacent level because of higher path loss as compared to the nodes located at the imaginary boundary.

In other words, the probability that a node becomes a member of the *m*th hop (i.e., the probability that it transmits in hop *m*) can be different for every single node. For example, a node present near the imaginary boundary of hop m−1 can become a part or member of the next hop *m*, given that it has not transmitted before and decoded the signal successfully in the next time slot. In Figure 11, some nodes present at imaginary boundary $s_{1}$ have high chances of being a member of either hop m−1 or hop *m*.

**Figure 13:**
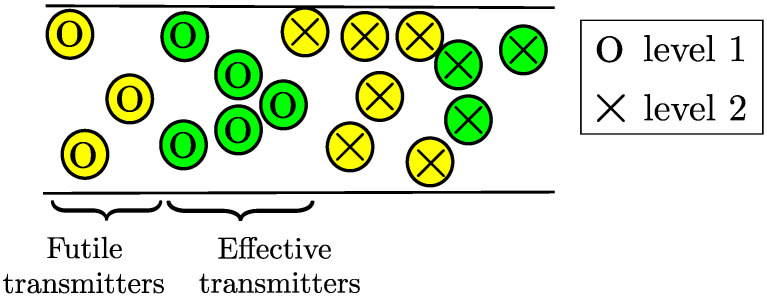
Futile and effective transmitters.

A system with OLA multi-hop transmissions can be energy-efficient, when the number of nodes of each level is only enough to successfully relay the message signal to the next level. In other words, in the high node density network, the transmissions from all the DF nodes of one level are not required for the formation of the next level. Those nodes, which are located near the source or near the boundary of prior level, have higher received power compared to the distant nodes. When these near nodes transmit to the nodes of next level, their transmissions have little or almost no effect on the decoding of next level nodes because of significantly high path losses. To allow only those nodes, which are near to the next level boundary, to transmit signal to the next level, while limiting those nodes that are near to source node, will conserve meaningful amount of energy. A criterion based on threshold is used to limit such nodes, where only the nodes whose received SNR levels are greater compared to a certain decoding threshold but smaller compared to an upper bound threshold are allowed to transmit. In Figure 13, nodes at any level are further divided into two sets of transmitters according to the above criteria: futile transmitters and effective transmitters. These two sets of transmitters may or may not be equal in size [45].

This network model is more practical compared to previous 2D models, because it reflects all the random properties of OLA networks such as node locations, level boundaries, and channel characteristics. Analysis of this network in [45] shows that the same network coverage can be obtained by using different network parameters, such as transmit power and average number of nodes in hops. That is, the network designer may choose any set of parameters depending on the network constraints and requirements. Furthermore, nodes in each level can be divided into active or passive transmitters to conserve a meaningful amount of energy of the system. The reliability, throughput, latency, average number of nodes in a level, and one-hop success all depend on transmit power, node density, distance between nodes, and network topology itself. A more detailed model for energy efficiency (EE) of OLA transmission with random geometry is studied in [64], where the authors adjust the degree of node participation to improve EE.

## 5. Multi-Packet OLA Transmission Networks

One major disadvantage of OLA or other CT schemes is increased interference by the group transmissions of multiple nodes. This interference can be categorized into two types: intra-flow and inter-flow. The intra-flow interference is caused by multiple packets from the same source, when the spatial separation between the co-channel packets, which propagate through the same multi-hop network but in different hops, is not large enough. Such inter-separation and the corresponding intra-flow interference level should be properly managed to optimized throughput. On the other hand, the second type, inter-flow interference, occurs by the packets originated from multiple sources. This inter-flow interference also needs to be considered in MAC protocol design to maximize the data rate of the entire network and address fairness between multiple users. For this reason, in this section, we consider theoretical models to optimize multi-packet OLA transmission, which are summarized in Table 2.

### 5.1. 1D Multi-Packet OLA Network

**Figure 14:**
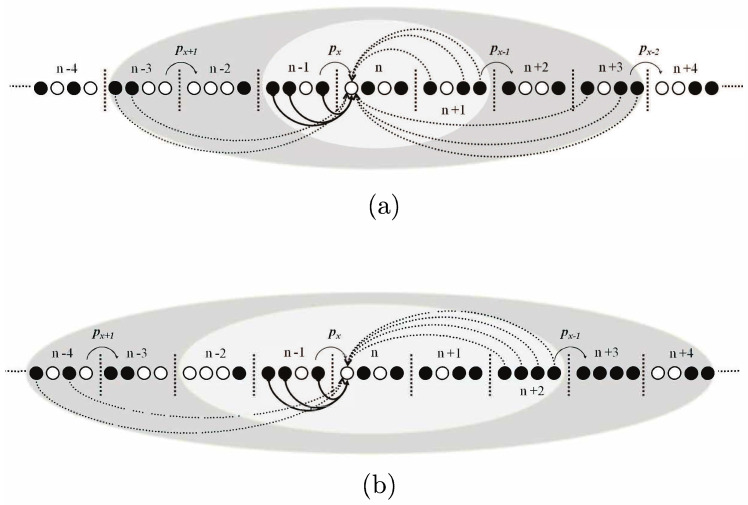
1D Muti-Packet OLA Network; (**a**) R=1, (**b**) R=2.

The two networks shown in Figure 14 are an extended version of non-overlapping 1D in [46], where levels are labeled from n−4 to n+4 and considered as a cluster. The hop occurs when nodes at any level forward the message signal to its adjacent levels. In this network topology, multiple packets are allowed to transmit simultaneously in a cooperative scenario in multi-hop transmission systems. Although simultaneous transmissions of multiple packets increase packet delivery rate, allowing multiple packets at the same time can cause interference between packets. There are two network parameters to be defined: (i) *R* is packet insertion rate (PIR) and defined as rate per time slot, at which point the source transmits a new packet, and (ii) *T* is the tiers of interference, which shows different number of levels interfere with the nodes of a level. At the top of Figure 14, R=1 means that source insert a packet after waiting one time slot, for instance, at level n−1, when DF nodes (the filled-black circles) transmit $p_{x}$ packet to the next adjacent level *n*, where *x* represents packet number being transmitted. In the same manner, DF nodes at level n+1 transmit $p_{x-1}$ to next adjacent level n+2 and so on. At the bottom of Figure 14, R=2 shows that the source transmits a packet after waiting two time slots between consecutive transmissions. That is, when level n−1 transmits $p_{x}$ to level *n*, level n+2 transmits $p_{x-1}$ to level n+3. Although the intended destination of level n−1 is level *n* for any value of *R*, it assumes omnidirectional antennas, which will interfere with other neighboring levels. The solid arrow lines from level n−1 to level *n* represent the desired signal of the fresh packet, whereas the dotted lines indicate the undesired signals from level n+1, n+3 and n−3, which affect the node at level *n* as shown in the figure. Different numbers of levels take part in interference with the nodes of a level depends on different tiers, *T*. For example, when T=1, undesired signals affect the node at level *n* coming from level n+1 only when R=1, as indicated by the white inner ellipse in the top figure of Figure 14. When T=2 and R=1, two more levels (i.e., n+3 and n−3) cause interference to the nodes of level *n*, as represented by the outer dark-gray ellipse.

For multiple flows of packets on a one-dimensional network, it is concluded that when we increase PIR, which means the network inserts a packet after waiting more time slots, the interference from other levels decreases because it offers larger path loss to the desired level and thus provides larger network coverage. In contrast, for lower values of *R*, which means more packets can be inserted simultaneously, throughput increases, and therefore delay would be less. In this context, the trade-off between the coverage and throughput needs to be exploited to obtain a certain quality of service.

### 5.2. 2D Multi-Packet OLA Network

**Figure 15:**
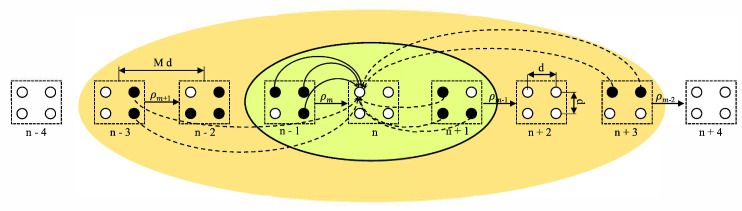
2D Multi-Packet OLA Network.

The network model in Figure 15 is almost the same as the previous model in Figure 14, but their dimensions are different. In the network topology, depicted in Figure 15, the nodes are placed in two dimensions, where levels are labeled with the indices from n−4 to n+4 and considered as cluster. The hop occurs when nodes at any level forward the message signal to its adjacent levels. The nodes are placed in 2D geometry of length *L* and width *H* as a square region. There are four nodes in each square region (cluster). In this network topology, multiple packets are allowed to transmit simultaneously in a cooperative scenario in multi-hop transmission system. Even though simultaneous transmission of multiple packets increases packet delivery rate, allowing multiple packets at the same time can cause interference between packets. As in the 1D multi-flow OLA network model, we have two key parameters: the PIR *R* and the tiers of interference *T*. In Figure 15, R=1 is considered, which implies that the source inserts a packet after waiting one time slot, for instance, at level n−1, when DF nodes (the black circles) transmit $p_{m}$ packet to next adjacent level *n*, where *m* represents packet number being transmitted. Similarly, DF nodes at level n+1 transmit $p_{m-1}$ to next adjacent level n+2 and so on. Although the intended destination of level n−1 is level *n*, it will also cause interference to other levels. The solid arrow lines from level n−1 to level *n* correspond to the desired signal, while the dotted lines indicate the undesired signals from levels n+1, n+3, and n−3, which interfere with the nodes at level *n* as illustrated in Figure 15.

Using this model, Reference [47] shows that the transmission rate increases with lower *R*, whereas the network coverage decreases. It is further proved that coverage can be improved by increasing the number of nodes and width. However, coverage may be reduced by increasing the length L because of higher average path loss. Therefore, there is again a trade-off between transmission reliability and latency of a network; the bigger hop will obtain for large value of *L*, and thus a lower number of hops will require reaching the destination but a larger number would be the average path loss of the system. For a smaller value of *L*, delay of the network will increase because shorter hops will be made, but it will give better reliability for each hop that occurs.

### 5.3. 2D OLA Network with Multiple Sources

**Figure 16:**
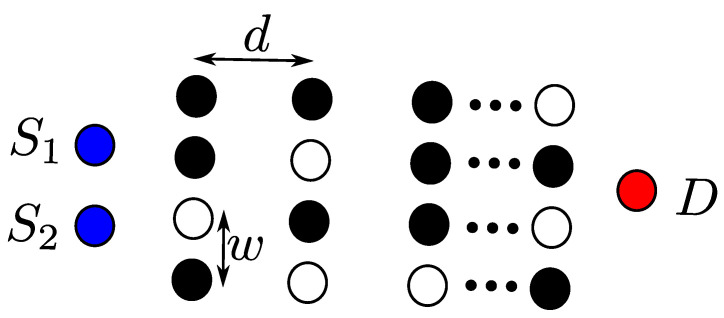
Muti-Source OLA model with nodes located in grid pattern.

In all the network topologies discussed above, a single flow is assumed with one source and destination pair. However, in reality, in WSNs, for example, any node (or sensor) can be a source, which starts transmission any time, when it has something to send. For this reason, in [48], the authors consider an OLA network model with multiple sources. Specifically, in this network topology, there are multiple sources that have independent information to be transmitted to a common distant destination (i.e., sink), as shown in Figure 16. Even though there are relays to transmit all of the sources’ information to the common destination, co-channel interference occurs, if the multiple sources transmit simultaneously using the same channel. A technique called linear network coding is used to avoid such interference in [48], in which intermediate nodes combine information from both sources for transmission. The intermediate relay nodes use the same mechanism of decode and forward at each hop.

**Figure 17:**
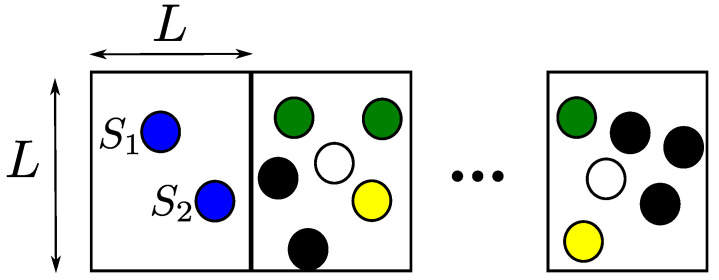
Muti-Source OLA model with randomly located nodes.

We can consider two network topologies, which correspond to Figure 16 and Figure 17, respectively. In the first topology, as illustrated in Figure 16, nodes are placed in regular pattern. On the other hand, the second topology in Figure 17 contains a fixed number of nodes, but the nodes are placed randomly in a strip-shaped network. In Figure 16, there are two sources, $S_{1}$ and $S_{2}$, which are located with a certain inter-separation, and destination node *D* is far away and common for these two sources. A group of *N* relay nodes are present to form a cluster, and nodes in the cluster are *d* distance away from the nodes of adjacent cluster and *w* distance away from nodes of same cluster. Further, it is assumed that both sources use the same transmit power $P_{t}$. The source nodes transmit their information signals using orthogonal channels or frequencies to avoid interference. Then, the intermediate nodes of the first cluster do DF to relay the information signal to the next adjacent nodes. The DF nodes in this network topology are those nodes that can decode information signals of both sources. Only the DF nodes will relay the information signal to the nodes of the next hop. The DF nodes in Figure 16 are represented by black circles, for instance, nodes 1, 2, and 4 in the first hop. Those nodes that either cannot decode any information signal or can decode only a single source information are not DF nodes, which are represented by white circles. At the physical layer, the information, which is decoded at relay node of both sources, is the combination of both sources. For example, $I_{1}$ and $I_{2}$ are two information messages from $S_{1}$ and $S_{2}$, respectively, and can be added at intermediate relay nodes and form a simple network code. These relay nodes of the first hop will then forward this codeword to the next adjacent nodes in the next hop. The nodes of the second hop receive multiple codewords from the previous nodes of the first hop and try to decode it. The nodes of the second hop are able to decode these codewords if and only if a node has received at least two codewords. The received codewords are further converted into new codeword, if successfully decoded.

In the second network’s topology, shown in Figure 17, there are two sources, while relay nodes are uniformly and randomly located in a square region of area L×L. In each hop, there are a fixed number *N* of relay nodes, which are randomly located. The network is fixed in the vertical dimension and extended in the horizontal dimension with contiguous square regions as shown in the figure. The transmission strategy is kept the same. The nodes in a certain OLA level receive independent messages from both sources and try to decode it, and then those nodes that decode messages of both sources successfully will make a codeword and then forward it to the next adjacent level. In this network topology, we assume that the nodes that decode either the message of $S_{1}$, $I_{1}$ or the message of $S_{2}$, $I_{2}$ will also forward their decoded message to the next level in the next hop. For instance, in the figure, the nodes represented by the black circles are DF nodes, which decode messages of both sources. In contrast, the white circles decode nothing, while the nodes indicated by the green circles decode $S_{1}$ message and the nodes represented by the green circles decode only $S_{2}$ message.

In the second and following hops, three groups of nodes (i.e., states 1, 2, and 3) send their messages. It is seen that, with time, more nodes could have decoded messages of both sources because nodes receive message from two disparate groups; either by those relay nodes that already have message of both sources or by those relay nodes that have message of a single source. The receiving relay nodes will obtain two disparate messages of both sources in this way; therefore, a greater number of relay nodes will obtain both messages of two sources. The number of relay nodes increases in state 2 with hop count until it achieves a maximum value.

## 6. Energy-Efficient OLA Protocols

In the IoT networks with small wireless devices, which have inherent power and hardware limitations, energy efficiency is considered to be one of the key design factors. The SNR advantage of the OLA can be used to provide energy-efficient transmission for applications with power and hardware limitations. In this section, we cover the energy-efficient OLA protocols.

### 6.1. OLA with Transmission Threshold (OLA-T)

The basic principle of OLA-T protocol is to limit the number of nodes that are retransmitting the source message. All the nodes that are nearer to the forward boundary and hardly decode the source message can take part in the creation of the next OLA. By “hardly decode”, we mean that the nodes are far from the source and have decoded the message in an acceptable manner. These nodes that receive the message with very high SNR do not participate in the creation of the next OLA. In other words, they do not transmit in the next time slot. The reason behind such selective membership is that they are nearer to the source, which suffer from high path attenuations. Thus, in the OLA-T protocols, a certain threshold is set such that the nodes that receive the source message with SNR values less than the threshold can transmit and be part of the next OLA [65]. This scheme can be applied to any OLA transmission scheme. OLA-T can save more than 50% of energy compared to the basic OLA with flooding. OLA with variable transmission threshold (OLA-VT) optimizes the thresholds as a function of level, which is also discussed in [20]. While OLA-T for disk-shaped networks is studied in [20], the authors of [66] consider OLA-T protocol for strip-shaped networks, which shows approximately 62% energy saving as compared to basic OLA. The same strip-shaped network is studied in [30], where OLA-T is applied for limited node density.

### 6.2. Alternating OLA-T (A-OLA-T)

A-OLA-T is an extended version of OLA-T, which has two sets of nodes. One of the sets includes the nodes that form OLA and take part in the broadcast of source message. The other set contains the nodes that do not at all transmit the message. The size and ratio of the two sets are subject to the SNR threshold for OLA-T. In the A-OLA-T protocol, on the other hand, non-transmitting nodes are also taken into consideration. That is, the nodes that do not transmit the message in the first broadcast cycle, will transmit in the next cycle; thus, an alternate set of nodes take part in each broadcast cycle. In this way, energy balancing between both sets can be achieved, and the energy of the entire network can be efficiently utilized. A-OLA-T increases network lifetime by approximately 17% as compared to basic OLA. Furthermore, the battery lifetime of the nodes in A-OLA-T become double as compared to the OLA-T nodes. The A-OLA-T protocols are optimized assuming a high node density in [67] by considering a disk-shaped network. In contrast, [68] investigates the A-OLA-T protocol for strip-shaped networks, in which the A-OLA-T shows the network-life extension by up to 278% relative to the Basic OLA.

### 6.3. OLA Concentric Routing Algorithm (OLACRA)

OLACRA is a multi-hop routing protocol with OLA transmissions, which are performed only for the nodes located in the upstream direction from the source towards the destination. The key factor in this protocol is the position of the nodes in the network and the knowledge of the upstream route from the source towards the destination. The nodes’ location can be identified by the carrier frequency method with the sink initializing the network by flooding in the entire network using either basic OLA or OLA-T [69].

### 6.4. OLA Concentric Routing Algorithm with Threshold (OLACRA-T)

OLACRA-T in [70] is an enhanced version of OLACRA, which makes further energy saving by allowing only the nodes near the upstream forward boundary to transmit. In OLACRA-T, nodes do not participate in an upstream transmission if their received signal power is greater than a certain threshold to adjust the degree of the node participation. 

## 7. Future Directions

OLA networks have shown to be of great interest for applications specific to low-power communications. Owing to the advantages of both array gain and diversity gain, the reliability issues in low-powered WSNs and IoT networks can be well addressed. However, there are still many challenges associated with these kinds of cooperative networks at both the algorithmic and application levels. In this section, we highlight some important areas where OLA and similar cooperative networks can be studied with different dimensions for their applications in real-time environments.

### 7.1. Scheduling in OLA Networks with Multiple Routes

Currently, studies of OLA are restricted to one route between either a source and destination or multiple sources and a single destination. However, in a dense OLA network, where a large number of nodes are present to communicate with each other, there may be instances where multiple routes between multiple source and destinations could be set up. However, scheduling at the physical or at the MAC layer becomes very necessary to avoid collisions between concurrent transmissions. More specifically, if some routes have *n* cooperators in each route and some have *m* cooperators, where a definite relationship between *n* and *m* is known or unknown, a scheduling strategy becomes inevitable and is an important direction to be investigated.

### 7.2. OLA and Cooperative Routing for Underwater Sensor Networks

Underwater sensor networks (UWSN) have been an area of prime interest in the recent past because of the advantages they offer. However, many algorithms have been carved out for routing the data in UWSN such as depth-based routing [71], directional flooding routing [72], focused beam routing [73], etc. The main concern in all of these protocols is that they are based on the concept of SISO communication, where one forwarder forwards the data from the source to the sink node where the sink node is generally present on the surface of water. Due to the inherent SISO issues such as low reliability and high outage probability, OLA CT networks with multiple forwarders pose an elegant alternative to overcome the aforementioned problems. However, even in the cooperative UWSN routing, the main issues handled are route selection, neighbor discovery, maintaining neighbor tables, and partner selection for one cooperator per hop [74,75,76]. In this kind of network, OLAs can be of considerable importance, where a wave of energy can travel from the source to sink without compromising on reliability and power. Therefore, the application of OLA CT networks for UWSN is of great importance and can be considered to improve the network performance.

### 7.3. Machine Learning and Deep Learning-Based Relay Selections in OLA Cooperative Networks

Recently, the use of machine learning (ML) and deep learning (DL) has been of great interest for various aspects of wireless communications and networks. On one hand, sophisticated ML-based algorithms are used for various resource management tasks in these systems to increase the performance, while on the other hand, they are used for various modern paradigms of edge computing and IoT networks. In cooperative IoT networks such as OLAs, an important issue is that of suitable relay selection such that various network metrics are satisfied. For instance, relay selection for energy-efficient operation may result in selecting different relays as compared to increasing the spectral efficiency. In OLAs, algorithms such as [77,78] can be effectively used to perform channel estimation, which can further be used for relay selection using ML- or DL-based algorithms. There are many recent works in this area such as [79,80,81], where various ML-based algorithms are used for relay selection in different environments. However, their applicability in OLA networks is an interesting aspect to investigate further [49,50].

### 7.4. Cooperation and Wireless-Powered Networks

An important feature of OLA networks in the energy-efficient operation where a very small energy is used to transmit data over large distances through diversity combining. Hence, OLAs are suitable for low-power applications such as swarm of robots in a factory or sensor networks in agricultural fields. However, these applications require network longevity, implying that the lifetime of the network should be as high as possible, owing to the fact that changing the batteries of these sensors is not possible on an intermittent basis. Therefore, OLA networks with amalgamation of energy harvesting also features an interesting dimension where a part of the incoming energy can be harvested for increasing the network lifetime, whereas the rest of the energy can be used for signal decoding/detection. Recently, References [82,83] have addressed a similar problem, where CT is used in energy-constrained devices for wireless-powered communications. The concepts can be further extended for OLA networks to study the effects of energy harvesting in each hop and the effects of cumulative energy savings can be studied in a multi-hop network.

### 7.5. Backscatter-Aided Cooperative Transmissions

Backscatter communication (BackCom) is another new paradigm, which has been introduced especially for low-powered networks. Although the concept of BackCom is not new and goes back to the development of RFID tags, which are commonly in practice these days, the concept of using backscatter with ambient sources is fairly new, where the sensors can be excited by any RF source present in the environment and can transmit their data to a central repository using direct or multi-hop communications. The concept of using cooperative relaying in BackCom networks is fairly novel and has been studied recently in many papers such as [84,85,86,87], where the relays use the backscatter concept to not only transmit data to next hops but also save energy by not consuming power of their own. OLA networks can benefit greatly from this concept, especially in low-powered sensor and actuator networks where both energy reliability are important.

### 7.6. Security for OLA Networks

CT and OLA transmissions are vulnerable to unfriendly wireless propagation. The reason is that when the message is transmitted from one level to another, there is a high chance of malicious nodes to corrupt the forwarded data, which may result in erroneous and even corrupt data to traverse the entire network. Although there have been studies such as [88,89] and the references therein that address the security issues for CT networks, most of the studies are limited to single or fewer hops in a uni-cast cooperative route. This may not be directly applied to OLA CT networks where the data must be propagated over multiple hops with finite boundaries and hence pose an important future research direction.

## 8. Conclusions

In this survey, we have presented an overview of existing OLA propagation scenarios and network models. These models will allow researchers to design new OLA-based protocols and evaluate their performances as 5G mMTC solutions without extensive simulation or experiments using test-bed. We have compared different models and provided guidelines to choose pertinent models for given network properties. Moreover, we have discussed possible future extensions of OLA techniques across various dimensions including scheduling multiple OLA routes, UWSN, ML-aided route optimization, wireless-powered networks, BackCom, and information security. In particular, it is crucial to optimize higher-layer protocols such as error control and MAC to handle a large number of simultaneous data flows in an energy-efficient fashion. In addition, OLA-based protocols should be tailored to specific application scenarios such as industrial IoT, smart healthcare, smart city, and autonomous vehicles.

## Figures and Tables

**Table 1:** Comparison of Different OLA Propagation Models with Single-Packet Transmission.

Reference	Shape	Density	Node Locations	Set Boundary
[20]	Strip (2D)	Infinite	Fixed	Deterministic
[14]	Disk (2D)	Infinite	Fixed	Deterministic
[37]	Linear (1D)	Finite	Fixed	Non-overlapping
[39]	Linear (1D)	Finite	Fixed	Overlapping
[40]	Linear (1D)	Finite	Fixed (collocated)	Non-overlapping
[41]	Linear (1D)	Finite	Random	Overlapping
[42]	Strip (2D)	Finite	Fixed	Overlapping
[43]	Strip (2D)	Finite	Random (fixed number)	Non-overlapping
[44]	Strip (2D)	Finite	Random (random number)	Non-overlapping
[45]	Strip (2D)	Finite	Random (random number)	Overlapping

**Table 2:** Comparison of Different OLA Propagation Models with Multi-Packet Transmissions.

Reference	Shape	Density	Node Locations	Set Boundary	Sources
[19]	Strip (2D)	Infinite	Fixed	Deterministic	1
[34]	Disk (2D)	Infinite	Fixed	Deterministic	1
[46]	Linear (1D)	Finite	Fixed	Non-overlapping	1
[47]	Strip (2D)	Finite	Fixed	Non-overlapping	1
[48]	Strip (2D)	Finite	Fixed	Non-overlapping	2 or more

## Abbreviations

The following abbreviations are used in this manuscript:

5G	Fifth Generation
B5G	Beyond Fifth Generation
IoT	Internet-of-Things
mMTC	massive machine-type communication
CT	Cooperative Transmission
OLA	Opportunistic Large Array
IoT	Internet-of-Things
mMTC	massive machine-type communication
eMBB	enhanced mobile broadband
D2D	Device-to-Device
VMISO	Virtual Multiple-Input-Single-Output
MISO	Multiple-Input-Single-Output
SISO	Single-Input–Single-Output
DF	Decode-and-Forward
AF	Amplify-and-Forward
MRC	Maximum Ratio Combining
EGC	Equal Gain Combining
SC	Selective Combining
MAC	Medium Access Control
1D	One-Dimensional
2D	Two-Dimensional
SDR	Software-Defined Radio
SNR	Signal-to-Noise-Ratio
PIR	Packet Insertion Rate
UWSN	Underwater Sensor Network
ML	Machine Learning
DL	Deep learning
BackCom	Backscatter Communication
